# Preoperative supine time for adrenal venous sampling: a prospective randomized controlled trial

**DOI:** 10.1186/s13063-023-07872-2

**Published:** 2024-01-02

**Authors:** Minzhi He, Yuhao Zhang, Xiaoxiao Song, Tianyue Zhang, Hailan Yu, Yongli Ji, Siyuan Gong, Peifei Chai, Jinyi Chen, Siwei Wang, Bing Chen, Xiaohong Xu, Zhenjie Liu

**Affiliations:** 1 Department of Vascular Surgery, The Second Affiliated Hospital, Zhejiang University School of Medicine, Zhejiang, 310009 Hangzhou China; 2 Department of Endocrine and Metabolic Diseases, The Second Affiliated Hospital, Zhejiang University School of Medicine, Zhejiang, 310009 Hangzhou China; 3 Assisted Reproduction Unit, Department of Obstetrics and Gynecology, Sir Run Run Shaw Hospital, Zhejiang University School of Medicine, Zhejiang, 310009 Hangzhou China; 4 Department of Anesthesiology, The Second Affiliated Hospital, Zhejiang University School of Medicine, Zhejiang, 310009 Hangzhou China; 5 Zhejiang University School of Medicine, Zhejiang, 310009 Hangzhou China

**Keywords:** Primary aldosteronism, Adrenal venous sampling, Supine time

## Abstract

**Background:**

Primary aldosteronism (P.A.) is the most common form of secondary hypertension, accounting for 5% of hypertensive patients and 17–23% of patients with resistant hypertension. Compared to primary hypertension, P.A. is more prone to cause severe organ damage and even early death. Adrenal venous sampling (AVS) is a practical confirmatory test for subtyping aldosterone-producing adenoma and bilateral adrenal hyperplasia, helping physicians to make an accurate decision between surgery or medication. According to guidelines, supine in bed before AVS is recommended for a desirable result of AVS. However, investigations about the most optimal preoperative supine time before AVS are lacking.

**Methods/design:**

This is a multi-center prospective randomized controlled study. One hundred twenty patients diagnosed as P.A. and willing for AVS examination will be included. Participants will be randomly allocated to a 15-min supine time group or 2-h supine time group. The primary outcome is the degree of biochemical remission (serum potassium and orthostatic ARR). The secondary outcomes are degrees of clinical remission (blood pressure, type and dose of antihypertensive drugs), the technical success rate, and the adverse event of AVS (selective index ≥ 2 is considered successful surgery without corticotropin stimulation).

**Discussion:**

P.A. is an intractable public health problem, and many techniques including AVS have been developed to identify this disease correctly. This study will help to understand whether the length of preoperative supine time would affect the diagnostic efficacy of AVS and thus help to formulate a more reasonable AVS procedure.

**Trial registration:**

ClinicalTrials.gov NCT05658705. Registered on 10 September 2022.

**Supplementary Information:**

The online version contains supplementary material available at 10.1186/s13063-023-07872-2.

## Introduction

### Background

Primary aldosteronism (P.A.) is a disease caused by excessive autonomous aldosterone from adrenal tumor or hyperplasia, characterized by the inhibition of the renin-angiotensin system, sodium retention, and excessive excretion of potassium and hydrogen [1]. P.A. was regarded as a rare disease since it was first discovered by Conn in 1955 [2] for it was found only in less than 1% of hypertensive patients [3]. However, recent studies have noticed that P.A. accounts for 5% of hypertensive patients in primary care [4], and the prevalence in patients with resistant hypertension has reached 17–23% [5]. A study on the Chinese population with newly detected hypertension also profiled at least 4% of patients suffered P.A [6]. Besides, P.A. is a significant cause of increased stroke risk, coronary artery disease, atrial fibrillation, ventricular hypertrophy, and renal damage [7]. It could be assumed that most P.A. patients missed proper diagnosis and suffered cardiovascular damage, which may explain the limitation of traditional hypertensive drugs on resistant hypertension.

P.A. includes two main subtypes: aldosterone-producing adenoma (APA, accounting for about 35%) and bilateral adrenal hyperplasia (BAH, accounting for about 60%). Other rare subtypes include primary adrenal hyperplasia, family aldosteronism, and aldosterone-secreting adrenocortical carcinoma [1, 8]. It is controversial whether P.A. patients should be treated with surgery or drugs [9]. Unilateral adrenalectomy is suggested for most patients with adenoma or primary adrenal hyperplasia, which statistically satisfied the goal of clinical and laboratory remission [10, 11]. However, anti-RAAS drugs and glucocorticoids are more recommended for BAH or family aldosteronism patients [12].

Raising the accuracy of diagnosis and subtyping is extremely important for P.A. patients. Screening of P.A. is based on the aldosterone to renin ratio (ARR), and people with abnormally elevated ARR should be screened by further oral salt loading or captopril confirmatory tests [13]. P.A. subtyping test is also an essential, although difficult and challenging, step [13]. Adrenal venous sampling (AVS) was now regarded as the gold standard for P.A. subtyping but only performed in limited centers [12, 14]. Our team was experienced in this technique. Recently, we have developed a computed tomography image fusion, coaxial guidewire technique, and fast intraprocedural cortisol testing (CCF) technique, which significantly increased the success rate of AVS to 98% and shortened the whole procedure time [15]. Moreover, we are still committed to exploring a more optimized AVS procedure.

AVS outcome was interpreted by comparing aldosterone levels in the adrenal venal of the dominant and nondominant side, which could be affected by the position change [16]. Before AVS without corticotropin injection, patients were suggested to keep a supine position to avoid aldosterone fluctuation [13, 17]. However, the length of supine time before AVS had no established standard, although authoritative guidelines recommend recumbency times ranging from 1 h to overnight [13, 17]. In previous work, we chose 1–2 h of supine time as the guideline suggested [17]. We observed many problems were caused by excessive supine time, such as patient’s urinary retention and anxiety, while reduced supine time was found to be more friendly for these patients.

The cortisol level of patients is also an important factor affecting the selective index (S.I.) during AVS. A cohort study showed that at least 15 min of rest before blood sampling was enough to minimize the stress effect and guarantee a satisfactory outcome [18]. Thus, we speculate that 15-min supine time is enough to minimize the fluctuation of aldosterone and cortisol fluctuation caused by position change and preoperative stress.

However, evidence of the most optimal supine time before AVS still lacks, which could simultaneously improve patient satisfaction and compromise the success rate. We expect that the shortened supine time will improve patient experience and ensure the success rate at the same time.

### Objectives

The primary aim of this study is to compare the effects of preoperative 15-min or 2-h supine to the efficacy of AVS

### Trial design

**Fig. 1:**
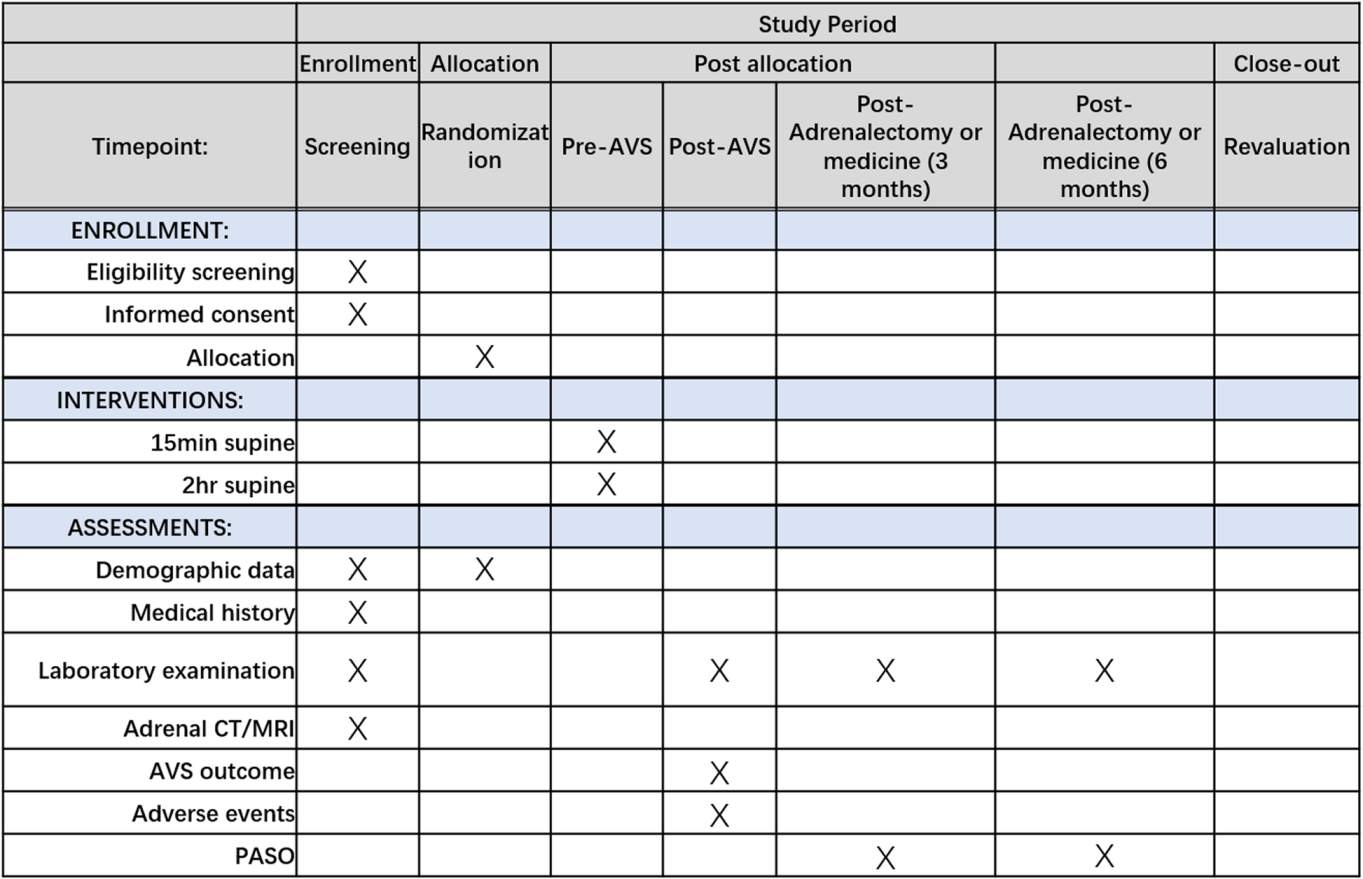
Standard Protocol Items: Recommendations for Interventional Trials (SPIRIT) enrollment schedule, interventions, and assessments

**Fig. 2:**
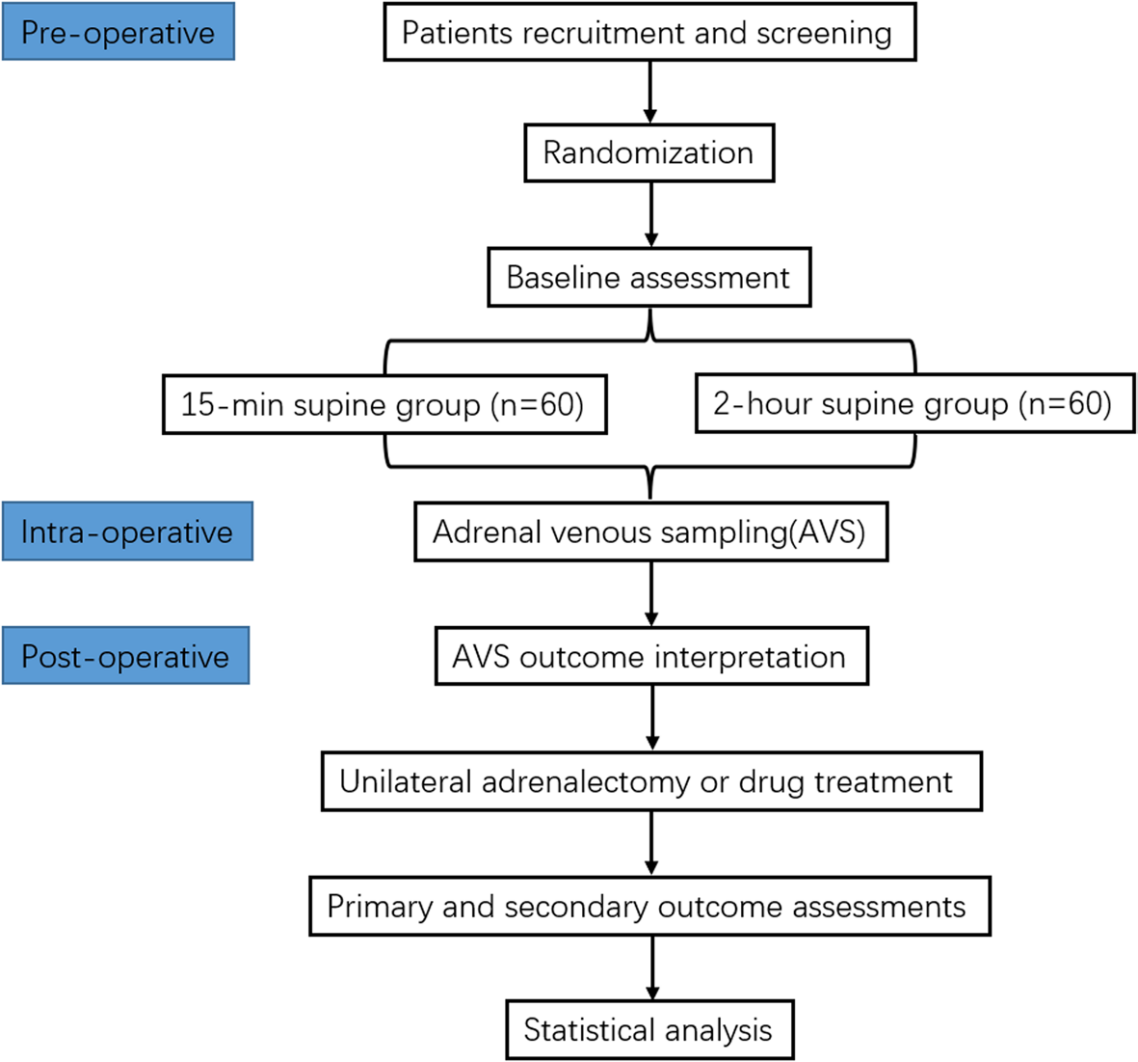
Flow chart of the trial

The study was designed as a multi-center, prospective, two-arm, parallel-group, randomized controlled trial. The protocol is reported in line with the Standard Protocol Items: Recommendations for Interventional Trials (SPIRIT) guidelines following the SPIRIT schedule (Fig. 1) and trial flow chart (Fig. 2). The study was registered on ClinicalTrials.gov: 05658705. The fundamental hypothesis is that reduced preoperative supine time did not affect the subtyping efficacy of AVS. Our hypothesis is based on a simple premise: 15-min preoperative supine time is enough to minimize the fluctuation of serum aldosterone and cortisol levels, thus maintaining the accuracy of AVS outcome.

## Methods

### Study setting

This study is taking place in three centers in China: The Second Affiliated Hospital of Zhejiang University of Medicine (initiation center), the First People’s Hospital of Jiashan (participating center), and the First People’s Hospital of Yuhang District of Hangzhou (participating center).

### Eligible participant

Participants are the patients considered P.A. and with willingness for AVS. Screening and preliminary classification of P.A. patients will be completed by endocrinology doctors, who will also be responsible for follow-up and medication adjustment. AVS and unilateral adrenalectomy will be carried out by a doctor of vascular surgery and urology.

The study was approved by the Ethics Committee of the Second Affiliated Hospital of Zhejiang University School of Medicine on 10/26/2022, before the trial registration on ClinicalTrials.gov.

The study will be carried out in accordance with the Helsinki Declaration. The inclusion, exclusion, and termination criteria for this trial are as follows:

#### Inclusion criteria

1. Patients are clinically diagnosed as primary aldosteronism with ARR≥37 (PAC showed as pg/ml, renin showed as μIU/mL) and passed through further P.A. confirmatory tests [13] (PAC-post CCT>110pg/ml, PAC-post SSIT >100pg/ml). PAC: plasma aldosterone concentration
2. Patients with willingness for AVS
3. Age 18 or above, male or female, with legal capacity

#### Exclusion criteria

1. Patients with suspected adrenocortical carcinoma or pheochromocytoma.
2. Patients do not accept or with a high risk of adrenal surgery.
3. Patients have been subtyping to glucocorticoid-suppressible hyperaldosteronism or familial hyperaldosteronism type III.
4. Patients were diagnosed with Cushing syndrome or subclinical Cushing syndrome.
5. Patients were treated with glucocorticoids recently.
6. Patients with whole body or venipuncture area infection.
7. Patients with venous access thrombosis.
8. Patients are allergic to iodine.
9. Patients with X-ray contraindications.
10. Patients with coagulation dysfunction.
11. Patients are unable to cooperate and follow up.

#### Termination criteria

1. Life-threatening events or other serious illnesses occurred during the trial.
2. Patients with incomplete basic information or be erroneously included.
3. Patients who dropped out, did not complete all treatment, or could not complete follow-up visits.

### Interventions

#### Description of the pre-, intra-, and postoperative management

Patient data will be collected in a case report form (CRF) designed by study staff during pre-, intra-, and postoperative courses and in 3 and 6 months after surgery or medicine therapy. All these data will be inputted into an electronic database for storage.

#### Participant enrollment and preoperative preparation

Patients will be initially included or excluded during hospitalization in the endocrinology department based on the patient’s basic information and medical history. The selected patients will be randomly divided into the 15-min supine group (experimental group) or the 2-h group (control group). Before surgery, patients will be informed of the risks, possible complications and expected examination results of AVS surgery, and signed informed consent.

Preoperative examination includes the following: (1) Adrenal enhanced CT: routine CT examination is performed to evaluate whether there are nodules, hyperplasia, and anatomical variation in bilateral adrenal glands; MRI examination was the alternative option for patients with renal insufficiency. (2) Necessary examination for surgery: complete blood count, serum chemistry profile, routine urinalysis, coagulation function, blood group, epidemic disease profile, electrocardiogram; (3) serum potassium, renin, and aldosterone.

Preoperative preparation includes the following: (1) replacing the drugs that may affect the serum renin and aldosterone, especially the mineralocorticoid receptor antagonist (MRAs); (2) keeping serum potassium in the normal range if possible (at least ≥ 3.0 mmol/l); (3) keeping blood pressure within the range of 140/100mmHg.

#### Adrenal vein sampling procedure

On the surgery day, surgeon and nurse will complete the double check, routine medical record, and signature. Patients of the 15-min group will be first sent to the operating room and kept supine before surgery, and femoral vein sheath inserting will be conducted at the end of supine time. During the operation of the 15-min group, the patient of the 2-h group will be sent to the waiting room and kept supine for 2 h. The surgery procedure is the same in both two groups, which were described below.

AVS without corticotropin stimulation was performed by two professional surgeons (Z.L. and M.H.) as previously described [15]. Briefly, a 5-F sheath was placed into the right common femoral vein under the local anesthetic. The left adrenal vein sampling was accomplished in the central adrenal vein by using a 4-F Simon 1 catheter and a 1.8-F microcatheter. The right adrenal vein was carried out by the guidance of coaxial guidewire technique by using a 4-F Simon 1 catheter. Digital subtraction venography helped to confirm the position of the catheters. The regular order of venous sampling was right adrenal vein, IVC, and left adrenal vein. Three milliliters of adrenal venous blood was obtained by intermittent gentle suction. Venous and IVC cortisol levels will be quickly measured by a conventional enhanced chemiluminescence immunoassay (ECLIA; Roche Diagnostics GmbH, Mannheim, Germany).

#### Postoperative management and follow-up

The result of AVS will be interpreted by an experienced doctor. Briefly, in this study, S.I. ≥ 2 indicates successful AVS, and L.I. ≥ 2 indicates unilateral primary aldosteronism. Unilateral P.A. patients will be suggested to accept the unilateral adrenalectomy, and bilateral adrenal hyperplasia patients will be recommended for medication therapy.

Patients will be followed up after unilateral adrenalectomy or optimized anti-hypertensive medicine therapy at 3 months and 6 months with clinical examination. All these data would be recorded in the PASO table, including the blood pressure, type, and dose of antihypertensive drugs, serum potassium, and orthostatic ARR.

#### Strategies to improve adherence to interventions

The investigators will inform the patients of the benefits and precautions of AVS surgery and thus build and maintain trust with patients.

#### Relevant concomitant care

To maintain the accuracy of AVS outcome, MRAs such as spironolactone were not permitted to be taken since 4 weeks before AVS. After AVS, for those who choose to be treated with medication, they will be instructed to take MRAs first, and if blood pressure is poorly controlled (SBP > 140 mmHg or DBP > 90 mmHg), they will be recommended to take MRAs combined with other antihypertensive drugs (ACEi/ARB or CCB will be firstly considered).

#### Provision for post-trial care

This trial’s target is to assess the effect of preoperative supine time for diagnostic efficiency of AVS. There is no anticipated compensation for trial participation.

### Outcomes

#### Basic information

Before treatment, researchers will collect basic characteristics (such as gender, age, education, marital status, height, and weight) and medical history (such as blood pressure, type and dose of antihypertensive drugs, serum potassium, orthostatic ARR) of recruited participants.

#### Primary outcome

1. Rate of biochemical remission

**Fig. 3:**
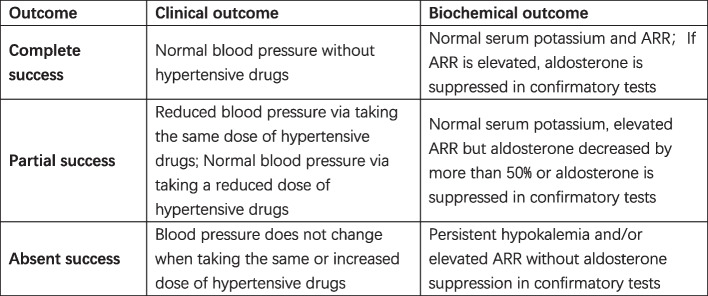
PASO table

Biochemical outcome evaluation in 3 and 6 months after AVS: serum potassium, orthostatic ARR. All these data will be recorded in the PASO table [11] (feasible criteria for the classification of outcomes of adrenalectomy for the treatment of unilateral primary aldosteronism). The detailed criteria of complete, partial, and absent biochemical remission are shown in Fig. 3.

#### Secondary outcome

1. Rate of clinical remission

Biochemical outcome evaluation in 3 and 6 months after AVS: blood pressure, types, and dose of antihypertensive drugs. All these data will be recorded in the PASO table. The detailed criteria of complete, partial, and absent clinical remission are shown in Fig. 3.

2. Successful catheterization rate

Intraoperative bilateral S.I. value was used to determine whether the blood collection cannula was successful. In the surgery without corticotropin, S.I. ≥ 2 was used as the standard for successful blood collection and L.I. ≥ 2 was used for judging the dominant side aldosterone secretion. S.I., selective index; L.I., lateralization index; Dom, dominant side; IVC, inferior vena cava; Nondom, nondominant side; PAC, plasma aldosterone concentration; PCC, plasma cortisol concentration. S.I. = PCC_side_/PCC_IVC_; L.I. = PAC_Dom_/PCC_Dom_:PAC_Nondom_/PCC_Nondom_.

3. Adverse events

Record the occurrence of adverse events, including adrenal venous hemorrhage and related adrenal insufficiency, hypertensive urgencies, anaphylactic shock, venous thrombosis, pulmonary embolism, etc.

### Participant timeline

The time schedule of this study, including enrolment, interventions, assessments, and visits for participants, is shown in Fig. 1.

### Sample size

The sample size calculation was performed using the PASS15 software, version 15.0.5, with reference to a prior randomized controlled trial that reported the value of targeting CXCR4 with 68Ga-Pentixafor PET/CT for subtyping P.A., which enrolled 120 cases [19]. According to this study protocol, a sample size of 60 participants in each group was calculated to sufficiently detect the target effect size (0.5) with a type I error of 5% (*α* = 0.05) and 90% power (*β* = 0.10) by the PASS15.0 software. Allowing for a 20% loss to follow-up, this study was designed to include 120 cases.

### Recruitment

Some efforts will be made to ensure enough participant enrollment in this study: more than 200 patients were eventually diagnosed as P.A. in our centers, and 100–120 AVS surgeries were performed each year in our centers with a success rate higher than 95%. Therefore, we think we need 1–1.5 years to enroll enough cases. Besides, we would implement a poster recruitment strategy in our hospital and on the Internet, which contains detailed information about this trial’s objective, contact method, and attention matters. Further, a specific member will help interested participants to complete registration for the trial.

### Assignment of interventions: allocation

#### Allocation-sequence generation

Random numbers within the range of 1 to 120 will be generated by an independent statistician using the SPSS V25.0 software. The allocation of participants will be determined based on the parity of the generated random numbers. Participants with an odd random number were assigned to the 15-min group, and participants with an even random number were assigned to the 2-h group.

#### Allocation—concealment mechanism

The result of random sequences will be placed into specific envelopes. These envelopes will be prepared by individuals who are not directly involved in the trial. After enrolled participants signed the informed consent, they will be given a sealed envelope to open.

### Assignment of interventions: blinding

This trial is open-label with blinded outcome assessors so unblinding will not occur. A third party will oversee the management to ensure the confidentiality of the study until the completion of the data analysis.

### Data collection and management

#### Plans for assessment and collection of outcomes

Basic information will be collected during the initial assessment and enrollment. Participants will complete and sign a questionnaire including basic characteristics (such as gender, age, education, marital status, height, and weight) and medical history. The data collection will be accomplished by a staff not involved in the study. Data will be collected at five time points: during screening, baseline, after AVS, at 3-month follow-up, and at 6-month follow-up. To ensure the data quality, all collected information will be securely stored in an electronic database, the access to which will be only restricted to specific statisticians. After 3 years, all data will be deleted.

#### Plans to promote participant retention and complete follow-up

To ensure the completion of the follow-up, all participants will be informed of the benefits and offered compensation for completing assessments. Regular phone reminders will be conducted during the follow-up period.

#### Confidentiality

All information and data can only be accessed by authorized accounts and authorization can only be granted by the chief investigator. Participants’ information will be encoded with a specific ID number to keep confidentiality. The database will be protected on a secured platform.

#### Additional consent provisions for collection and use of participant data and biological specimens

No biological specimens will be collected in this study.

### Statistical analysis

An independent statistician will complete the statistical analysis of the study result via the SPSS V25.0. Baseline characteristics will be described by descriptive statistics. *x̅* ± *s* will represent continuous variables with the normal distribution, and the *t* test will compare the differences between the two groups. The Mann–Whitney *U* test will be used in continuous variables that were not normally distributed. Categorical variables will be expressed as frequency (%), and the chi-square test will be used to compare the differences between the two groups. Effect estimates will be provided with corresponding 95% confidence intervals, and *P* values ≤ 0.05 will be considered statistically significant. All randomized participants will be included in the analyses, and data will be analyzed in the group where the participants were randomized initially. The experimental group includes the available data from the 15-min supine group, and the control group includes the available data from the 2-h supine group. The principles of intention-to-treat analysis will be used to preserve the unique benefit of randomization, regardless of whether that participant received the allocated intervention. Multiple imputation (MI) using the mice package in R will be applied to analyze the missing data.

Further secondary analyses will include pre-specified subgroup analyses according to age and the two stratification/minimization variables, investigated by introducing the relevant interaction with treatment allocation into the regression model. In addition, to assess the stability of any intervention effect, we will fit a mixed model for the primary outcome at 3 and 6 months, adjusted for baseline measures.

### Oversight and monitoring

#### Coordinating center and trial management committee

There are 3 centers that participated in the study: the Second Affiliated Hospital of Zhejiang University of Medicine (initiation center), the First People’s Hospital of Jiashan (participating center), and the First People’s Hospital of Yuhang District of Hangzhou (participating center). The management committee consisted of chief investigator, principal investigators of each workstream, statisticians, and so on and will monitor the process of recruitment, study management, and implementation interventions.

#### Adverse event reporting and harms

This study identifies adverse events including adrenal venous hemorrhage and related adrenal insufficiency, hypertensive urgencies, anaphylactic shock, venous thrombosis, and pulmonary embolism. We will report adverse events to the DMC (Data Monitoring Committee) within 24 h. The reporting will include details on severity, occurrence time, duration, actions taken, outcomes, and causality assessment.

#### Trial conduct auditing

All participating members including doctors, nurses, and analysts will be trained to get acquainted with the whole procedure of this study. Regular online meetings will be held weekly to address difficulties. An independent DMC will be instituted to oversee trial data and adverse reactions and provide suggestions. Monthly meetings will be convened to monitor the trial progress, safety problems, and data quality.

#### Protocol amendments

The principal investigator will be responsible for all protocol modifications. Any updated protocols will be promptly reported to the Chinese Clinical Trial Registry, Clinical Trials, institutional review board (IRB), and all other trial participants.

#### Dissemination plans

The public will not directly participate in this study. The data will not be released directly to the public. The protocol of this study will be publicly available, and the results will be authored and published in academic journals.

## Discussion

P.A. is commonly discovered among patients with resistant hypertension, with a higher risk for earlier cardiac disease, peripheral arterial disease, stroke, and renal injury [20]. With the advancement of diagnosis and subtyping method, P.A. is gradually getting attention from medical staff [21]. However, many controversies remain about the subtyping diagnosis and subsequent treatment of P.A.

C.T. imaging or MRI had limited diagnostic power in P.A. subtyping. AVS is now regarded as an essential standard tool for screening unilateral primary aldosteronism, due to its high efficacy in distinguishing no functional incidentalomas from aldosterone-producing adenomas [13]. The Endocrine Society recommends that all patients with hypertension suspected as primary aldosteronism should accept AVS examination [13]. However, there are many difficulties in performing AVS and interpreting the outcome, and only a few centers can and are willing to carry out this examination. One of the reasons is that surgical protocols of AVS are very different between medical centers, making it hard to standardize the AVS procedure and thus affect the success rate. On the other hand, the patient’s feelings were lack of attention during the whole procedure, which is also an important part of medical quality.

The outcome of AVS, including the selective index (S.I.) and lateralization index (L.I.), are calculated by the serum cortisol and aldosterone levels sampled in the adrenal and inferior veins. In the recent guideline, the European Society of Hypertension recommended S.I. > 2 to demonstrate correct cannulation, and L.I. > 4 to identify the unilateral P.A [22]. Thus, the fluctuation of cortisol and aldosterone is disastrous for interpreting AVS results [23]. Investigators have raised several approaches to avoid the deviation, including corticotropin application and long-time enforced recumbency [17], although there is still a lack of a standardized protocol for AVS.

In this study, we planned to pay attention to the supine time before AVS, which was currently an underappreciated part of the AVS procedure. In current clinical guidelines, the preoperative supine time was set to range from 1 h to overnight [13, 17]. Indeed, long-time supine time, in terms of result, reduced aldosterone fluctuations caused by position change and increased the accuracy of AVS results [16]. However, we should be aware that some patients will feel intense or anxious with time prolonging which may affect the cortisol level and that excessive time reduces the efficiency of surgery [18, 24]. Thus, preoperative supine is important to guarantee a satisfactory outcome of AVS, but the time length of the supine position should be reduced to take care of the patient's feelings.

Our study is the first clinical trial focused on the preoperative lying time of AVS. One limitation of this trial is that it is a multi-center study carried out only in centers in China. Given the differences in the management and success rate of AVS between different centers, this may affect the generalizability of this research. Another limitation of this study is that the patients and investigators cannot be blinded due to the type of study. To minimize the potential influence of this limitation, we will assign the most professional vascular doctor to perform the same operation on the two groups of patients, and the final statisticians will be blind.

In summary, this study will compare the effect of preoperative long-term supine to short-term supine on the outcome of AVS, thereby optimizing effective AVS surgery procedure which would satisfy both the patients and physicians.

## Trial status

This protocol is registered at ClinicalTrials.gov under the number NCT05658705 and it was first posted on 12/21/2022 and last updated on 27/08/2023, https://www.clinicaltrials.gov/ct2/show/NCT05658705. Recruitment will take place from 03/01/2023 to 03/01/2025.

### Supplementary Information

**Additional file 1.**

## Data Availability

Not applicable.

## Abbreviations

P.A.: Primary aldosteronism
AVS: Adrenal venous sampling
BMI: Body mass index
SPIRIT: Standard Protocol Items: Recommendations for Interventional Trials
MRA: Mineralocorticoid receptor antagonist

## References

[1] Zennaro MC, Boulkroun S, Fernandes-Rosa FL (2020). Pathogenesis and treatment of primary aldosteronism. Nat Rev Endocrinol..

[2] Conn JW (1955). Presidential address. I. Painting background. Ii. Primary aldosteronism, a new clinical syndrome. J Lab Clin Med..

[3] Kaplan NM (1967). Hypokalemia in the hypertensive patient, with observations on the incidence of primary aldosteronism. Ann Intern Med..

[4] Rossi GP, Bernini G, Caliumi C, Desideri G, Fabris B, Ferri C, Ganzaroli C, Giacchetti G, Letizia C, Maccario M, Mallamaci F, Mannelli M, Mattarello MJ, Moretti A, Palumbo G, Parenti G, Porteri E, Semplicini A, Rizzoni D, Rossi E, Boscaro M, Pessina AC, Mantero F (2006). A prospective study of the prevalence of primary aldosteronism in 1,125 hypertensive patients. J Am Coll Cardiol..

[5] Calhoun DA (2007). Is there an unrecognized epidemic of primary aldosteronism?. Pro Hypertension..

[6] Xu Z, Yang J, Hu J, Song Y, He W, Luo T, Cheng Q, Ma L, Luo R, Fuller PJ, Cai J, Li Q, Yang S (2020). Primary aldosteronism in patients in China with recently detected hypertension. J Am Coll Cardiol..

[7] Savard S, Amar L, Plouin PF, Steichen O (2013). Cardiovascular complications associated with primary aldosteronism: a controlled cross-sectional study. Hypertension..

[8] Vaidya A, Mulatero P, Baudrand R, Adler GK (2018). The expanding spectrum of primary aldosteronism: implications for diagnosis, pathogenesis, and treatment. Endocr Rev..

[9] Young WF (2019). Diagnosis and treatment of primary aldosteronism: practical clinical perspectives. J Intern Med..

[10] Sukor N, Kogovsek C, Gordon RD, Robson D, Stowasser M (2010). Improved quality of life, blood pressure, and biochemical status following laparoscopic adrenalectomy for unilateral primary aldosteronism. J Clin Endocrinol Metab..

[11] Williams TA, JWM L, Mulatero P, Burrello J, Rottenkolber M, Adolf C, Satoh F, Amar L, Quinkler M, Deinum J, Beuschlein F, Kitamoto KK, Pham U, Morimoto R, Umakoshi H, Prejbisz A, Kocjan T, Naruse M, Stowasser M, Nishikawa T, Young WF, Gomez-Sanchez CE, Funder JW, Reincke M (2017). Outcomes after adrenalectomy for unilateral primary aldosteronism: an international consensus on outcome measures and analysis of remission rates in an international cohort. Lancet Diabetes Endocrinol..

[12] Reincke M, Bancos I, Mulatero P, Scholl UI, Stowasser M, Williams TA (2021). Diagnosis and treatment of primary aldosteronism. Lancet Diabetes Endocrinol..

[13] Funder JW, Carey RM, Mantero F, Murad MH, Reincke M, Shibata H, Stowasser M, Young WF (2016). The management of primary aldosteronism: case detection, diagnosis, and treatment: An endocrine society clinical practice guideline. J Clin Endocrinol Metab..

[14] Zhong S, Zhang T, He M, Yu H, Liu Z, Li Z, Song X, Xu X (2022). Recent advances in the clinical application of adrenal vein sampling. Front Endocrinol (Lausanne)..

[15] Liu Z, He M, Song X, Xu F, Zhang B, Chen B, Yu P, Zhou H, Shan L, Wang H, Gu Z, Zhong S, Xu X, Tao Z, Chen B, Gu W (2021). Computed tomography image fusion, coaxial guidewire technique, fast intraprocedural cortisol testing technique improves success rate and decreases radiation exposure, procedure time, and contrast use for adrenal vein sampling. J Hypertens..

[16] Te Riet L, van Esch JH, Roks AJ, van den Meiracker AH, Danser AH (2015). Hypertension: renin-angiotensin-aldosterone system alterations. Circ Res..

[17] Rossi GP, Auchus RJ, Brown M, Lenders JW, Naruse M, Plouin PF, Satoh F, Young WF (2014). An expert consensus statement on use of adrenal vein sampling for the subtyping of primary aldosteronism. Hypertension..

[18] Seccia TM, Miotto D, Battistel M, Motta R, Barisa M, Maniero C, Pessina AC, Rossi GP (2012). A stress reaction affects assessment of selectivity of adrenal venous sampling and of lateralization of aldosterone excess in primary aldosteronism. Eur J Endocrinol..

[19] Zheng Y, Long T, Peng N, Zhen M, Ye Q, Zhang Z, et al. The value of targeting cxcr4 with 68ga-pentixafor pet/ct for subtyping primary aldosteronism. J Clin Endocrinol Metab. 2023:dgad421. 10.1210/clinem/dgad421 37477496

[20] Douma S, Petidis K, Doumas M, Papaefthimiou P, Triantafyllou A, Kartali N, Papadopoulos N, Vogiatzis K, Zamboulis C (2008). Prevalence of primary hyperaldosteronism in resistant hypertension: a retrospective observational study. Lancet..

[21] Funder JW (2019). Primary aldosteronism. Hypertension..

[22] Mulatero P, Sechi LA, Williams TA, Lenders JWM, Reincke M, Satoh F, Januszewicz A, Naruse M, Doumas M, Veglio F, Wu VC, Widimsky J (2020). Subtype diagnosis, treatment, complications and outcomes of primary aldosteronism and future direction of research: a position statement and consensus of the Working Group on Endocrine Hypertension of the European Society of Hypertension. J Hypertens..

[23] Betz MJ, Zech CJ (2022). Adrenal venous sampling in the diagnostic workup of primary aldosteronism. Br J Radiol..

[24] Law R, Clow A (2020). Stress, the cortisol awakening response and cognitive function. Int Rev Neurobiol..

